# Feasibility and acceptability of an in-home digital device health and activity assessment platform in a diverse South Texas cohort: a pilot study

**DOI:** 10.3389/fdgth.2025.1603062

**Published:** 2025-09-01

**Authors:** Julia J. Mathews, Rachel Mulavelil, Nathaniel Rodrigues, Tiffany F. Kautz, Kevin Cosgrove, Chen-Pin Wang, Daniel MacCarthy, Roman A. Fernandez, Sarah Gothard, Nicole Sharma, Luis Serranorubio, Vanessa M. Young, Lyndsey Miller, Sudha Seshadri, Jeffrey Kaye, Zachary T. Beattie, Mitzi M. Gonzales

**Affiliations:** ^1^ Glenn Biggs Institute for Alzheimer’s & Neurodegenerative Diseases, University of Texas Health Science Center, San Antonio, TX, United States; ^2^ Department of Neuroscience, University of Virginia, Charlottesville, VA, United States; ^3^ Oregon Alzheimer’s Disease Research Center, Oregon Health & Science University, Portland, OR, United States; ^4^ Oregon Center for Aging & Technology, Oregon Health & Science University, Portland, OR, United States; ^5^ Graduate School of Biomedical Sciences, University of Texas Health Science Center, San Antonio, TX, United States; ^6^ Department of Neurology, Boston University School of Medicine, Boston, MA, United States; ^7^ Department of Neurology, Cedars-Sinai Medical Center, Los Angeles, CA, United States

**Keywords:** aging, cognition, daily life, digital biomarkers, older adults, technology, wearables

## Abstract

**Introduction:**

Health tracking technologies hold promise as a tool for early detection of cognitive and functional decline.

**Methods:**

This pilot study of 5 households [*N* = 7 residents, mean age: 74 (5), 71% Hispanic, 14% Black] used the Oregon Center for Aging & Technology (ORCATECH) platform to evaluate the technology and acceptance of the technology over a one-year interval in South Texas. Cognitive assessments and other surveys were administered at baseline and end-of-study visits.

**Results:**

Participants felt comfortable with the technology in their homes (86% Very Satisfactory or Satisfactory) and did not express privacy concerns (100% Very Satisfactory or Satisfactory).

**Conclusion:**

Health, cognition, and activity measures did not significantly change from baseline to end-of-study. Depression scores significantly improved (*p* = 0.034). The ORCATECH platform was an acceptable method of analyzing health and activity in a small, but diverse older population.

## Introduction

Non-Hispanic Black and Hispanic adults are at a higher risk of cognitive decline and dementia compared to non-Hispanic Whites. Nonetheless, they remain underrepresented in research on Alzheimer's disease and related dementias (ADRD). Early detection of cognitive decline using biomarkers is essential for prevention and intervention efforts, especially considering recent advances in disease modifying therapies (1, 2).

Digital biomarkers provide an opportunity to continuously capture data relevant to cognition and daily functioning. The Oregon Center for Aging & Technology (ORCATECH) platform, which assesses functional change at home, has been developed over the last two decades and includes wearables, in-home sensors, and other devices to collect data relevant to multiple domains of functioning (3, 4). Beattie et al*.* demonstrated the platform to be a reliable method of collecting health data (5). However, research on user experiences in diverse populations is necessary to tailor digital health solutions to communities that have not been adequately represented in this line of research (6). Feasibility studies offer valuable insights for evaluating new technologies before larger implementation trials, particularly in underrepresented populations where technology acceptance may differ.

We sought to assess the acceptability of the ORCATECH platform in the diverse San Antonio, South Texas population. As a secondary aim, we evaluated changes in health and activity levels using the ORCATECH platform.

## Methods

### Participants

Participants were recruited between May 2021 and July 2021 through community outreach events and contact with previous research participants at the Glenn Biggs Institute in San Antonio, Texas. Eligible participants were 62 years or older, lived in multi-room residences with reliable internet, had basic computer/email experience, and resided alone or with one other adult. Exclusion criteria included significant mobility limitations, uncontrolled medical conditions preventing study completion, household resident size >2, or an inability to provide informed consent independently. The study was approved by the IRB at UT Health San Antonio. All study participants signed the written informed consent.

### Technology

Required technologies included passive infrared motion sensors, door contact sensors, and an actigraphy watch. Device installation protocols were derived from Beattie et al. (5) and conducted during standardized home visits within four weeks of enrollment. Trained research staff conducted all installations within four weeks of enrollment and provided comprehensive orientation sessions for each device. Participants received detailed contact information for technical support and were encouraged to report any device malfunctions immediately. The monitoring period lasted twelve months following device installation.

### Movement detection devices

Our installation team deployed a standardized sensor array in each home, beginning with passive infrared motion sensors (NYCE Control, Burnaby, BC, Canada) strategically placed in every room to capture general activity patterns. Door contact sensors from the same manufacturer were mounted on all exterior doors to monitor home exits and entries, providing insights into community engagement and daily routines.

### Health activity monitoring devices

Each participant received an activity tracking watch (Withings Steel, Withings, Issy-les-Moulineaux, France) configured to monitor step count, activity levels, and sleep patterns without requiring participants to download or manage mobile applications. This device served as our primary wearable sensor for capturing both in-home and community-based activities.

Beyond these required components, participants could select from several optional monitoring devices based on their preferences and comfort level. An electronic pillbox (TimerCap, Moorpark, CA, USA*)* was used to analyze participants' pill-taking routines (4 of 7 participants accepted). An electronic bed mat (Emfit Quantified Sleep; Emfit Ltd, Vaajakoski, Finland) was used to detect heart rate, respiratory rate, and hours of sleep (6 of 7 participants accepted). Finally, a digital scale (Withings Body Cardio digital scale; Withings, Issy-les-Moulineaux, France) was used to measure participants' weights on a weekly basis (all 7 participants accepted).

### Annual study visits

At in-person baseline and 12-month end-of-study visits, participants completed the Uniform Data Set version 3 (UDS-3) National Alzheimer's Coordinating Center (NACC) clinical assessment (7), which includes a standardized neuropsychological battery (8).

### Surveys

Participants completed weekly Qualtrics surveys online that inquired about mood, changes in weekly routines, home visitors, extended home absences (e.g., vacation), and other relevant questions to gauge health and activity (9–11). If a participant did not complete the survey within three days, a reminder was emailed. At end-of-study, participants completed a user experience survey designed to elicit their opinions on the devices and their overall participation.

### Data analysis

We used SAS to conduct all analyses, while figures were generated using GraphPad Prism 10. Our analytical approach reflected the study's feasibility objectives, emphasizing descriptive statistics appropriate for this preliminary investigation. We calculated descriptive statistics of the health and wellness domains at three time periods: baseline (45 days, with the exception of the initial 2-week installation period), midpoint (days 151–210), and study end (the final 60 days).

For participant-level analyses, means and standard deviations of daily or weekly (scale only) measurements within each time period were calculated. For data that was not collected daily, the weekly statistics were prorated if a minimum of 2 measurements were available. Device data completeness was measured as the percentage of days or weeks during which device data was not missing. The overall mean of the study sample within a time interval of interest was the average of participant-level means, and the overall variability of the study sample was the squared root of the pooled variance (i.e., average of participant-level variance within the time interval of interest). Given our small sample size and feasibility study design, we interpreted all results as preliminary and hypothesis-generating.

## Results

### Participants demographics

Seven participants [mean age: 74 (SD = 5) years, 71% Hispanic, 14% Black] were enrolled in the study (Table 1). Two households consisted of a male and female couple, while the remaining three households were single females.

**Table 1:** Study demographics.

Baseline participant characteristics (*N* = 7)		
Number of residences, *N*		5
Lives alone, *N* (%)		3 (43)
Education, Mean years (SD)		16.7 (3.4)
Age, Mean years (SD), range		74 (5), 65–80
Female, *N* (%)		5 (71)
Race, *N* (%)	Black	1 (14)
	White	6 (86)
Ethnicity, *N* (%)	Hispanic	5 (71)
	Non-Hispanic	2 (29)
UDS-3 cognition and depression scores	Baseline, *N* = 7	Follow-Up, *N* = 7
MoCA, mean (SD)^a^	25 (2)	26 (2), *p* = 0.2
CDR sum, mean (SD)^a^	0.57 (0.73)	0.50 (0.77), *p* > 0.9
CDR global = 0, *N* (%)	5 (71)	5 (71)
CDR global = 0.5, *N* (%)	2 (29)	2 (29)
GDS-15, mean (SD)^a^	6 (2)	1 (2), *p* = 0.034

^a^ Wilcoxon signed-rank tests were used to determine significant differences between baseline and follow up.

### End-of-study user experience

**Figure 1:**
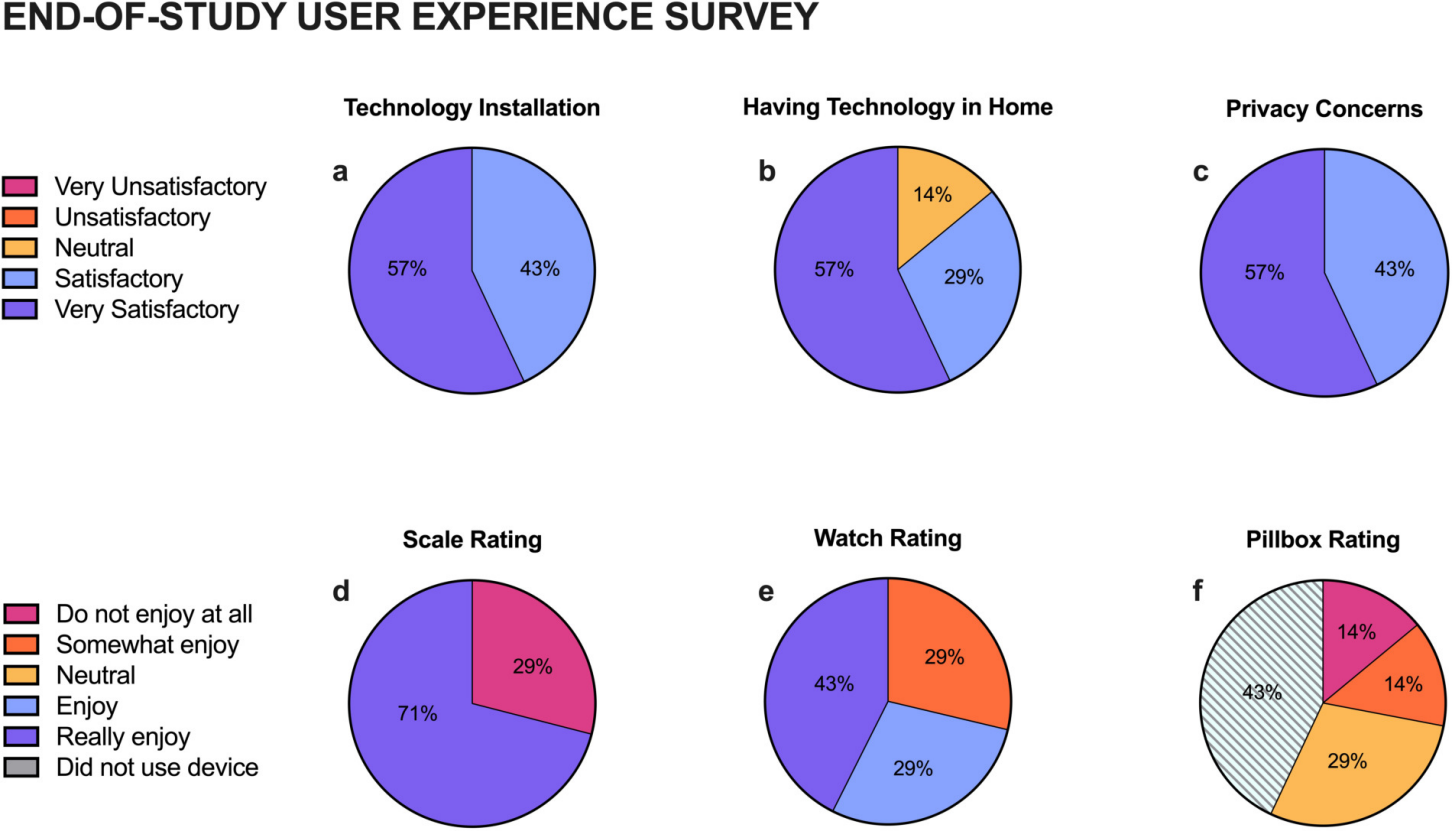
End-of-Study user experience survey results. Participants rated their satisfaction of the technology installation process **(a)**, having technology in their home **(b)**, and privacy **(c)**, as well as how much they enjoyed the usable devices: scale **(d)**, watch **(e)**, and pillbox **(f)**.

All participants completed the end-of-study survey. Participants were satisfied with the installation process (100% Very Satisfactory and Satisfactory) and the technology platform (86% Very Satisfactory or Satisfactory). Concerns related to privacy were adequately addressed (100% Very Satisfactory or Satisfactory) (Figures 1a–c). Participants generally rated the scale (71% Enjoyed) and watch (71% Enjoyed) positively but gave the pillbox neutral to low ratings (49%) (Figures 1d–f).

### Participant cognition, survey, and activity results

**Figure 2:**
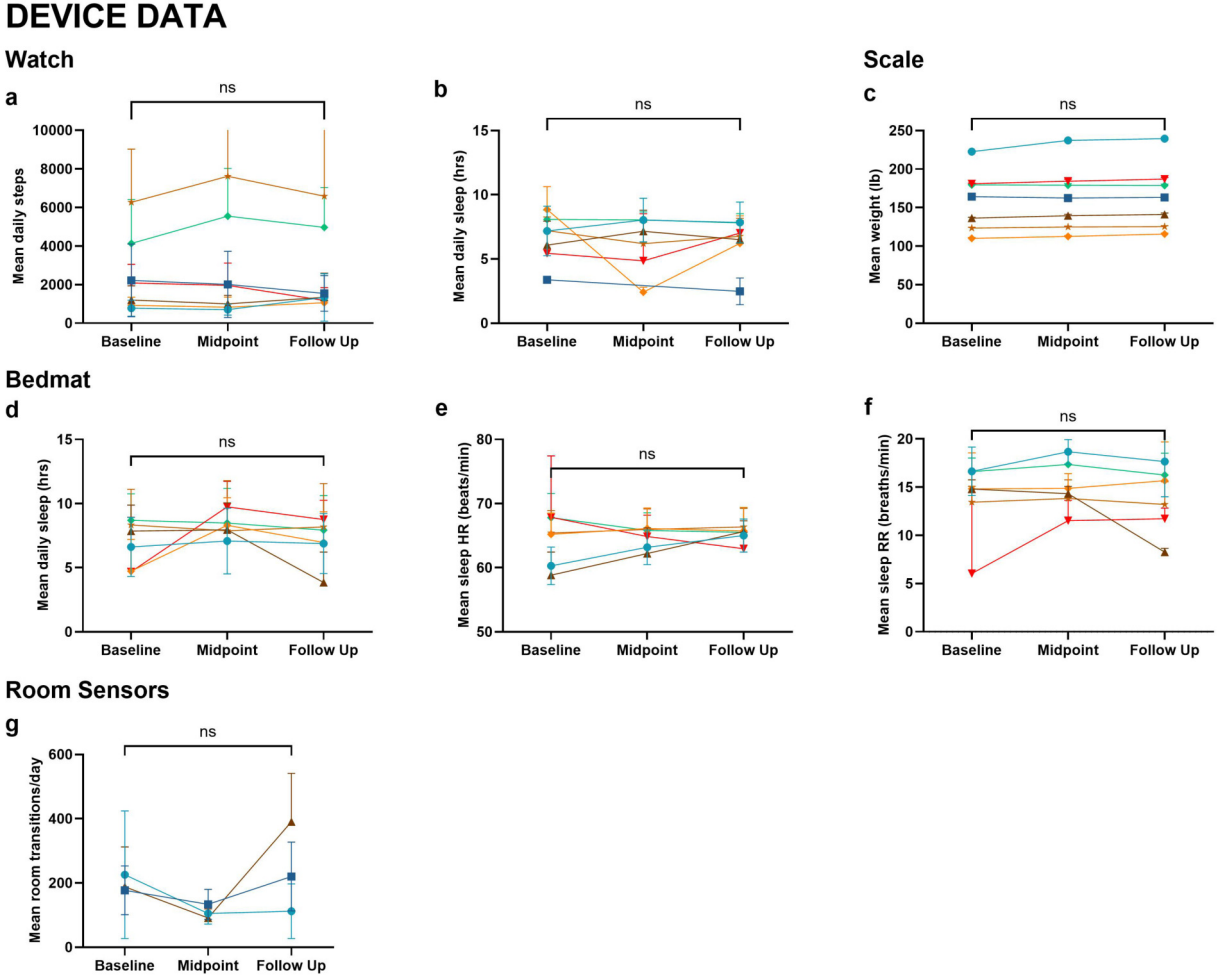
Device data. Metrics collected from wearable and in-home devices across Baseline, Midpoint, and Follow-Up include: **(a)** Mean daily steps (watch); **(b)** Mean daily sleep duration (watch); **(c)** Mean weight (scale); **(d)** Mean daily sleep duration (bedmat); **(e)** Mean sleep heart rate (bedmat); **(f)** Mean sleep respiratory rate (bedmat); **(g)** Mean room transitions per day (room sensors). Wilcoxon signed-rank tests were used to compare baseline and follow-up measures. No statistically significant differences were observed (ns). Each line represents an individual participant, with color coding consistent across figures.

At baseline, 5 participants (71%) had a global Clinical Dementia Rating (CDR) score of 0 and two (29%) had a global CDR of 0.5 with no changes occurring at the one year-follow-up. Montreal Cognitive Assessment (MoCA) scores also did not significantly change over the study period. However, depression scores, as measured by the Geriatric Depression Scale 15-Item (GDS-15), significantly improved (*p* = 0.034) (Table 1). There were no significant changes in activity levels as measured by the devices (Figure 2).

### Participant device and survey completion rates

**Figure 3:**
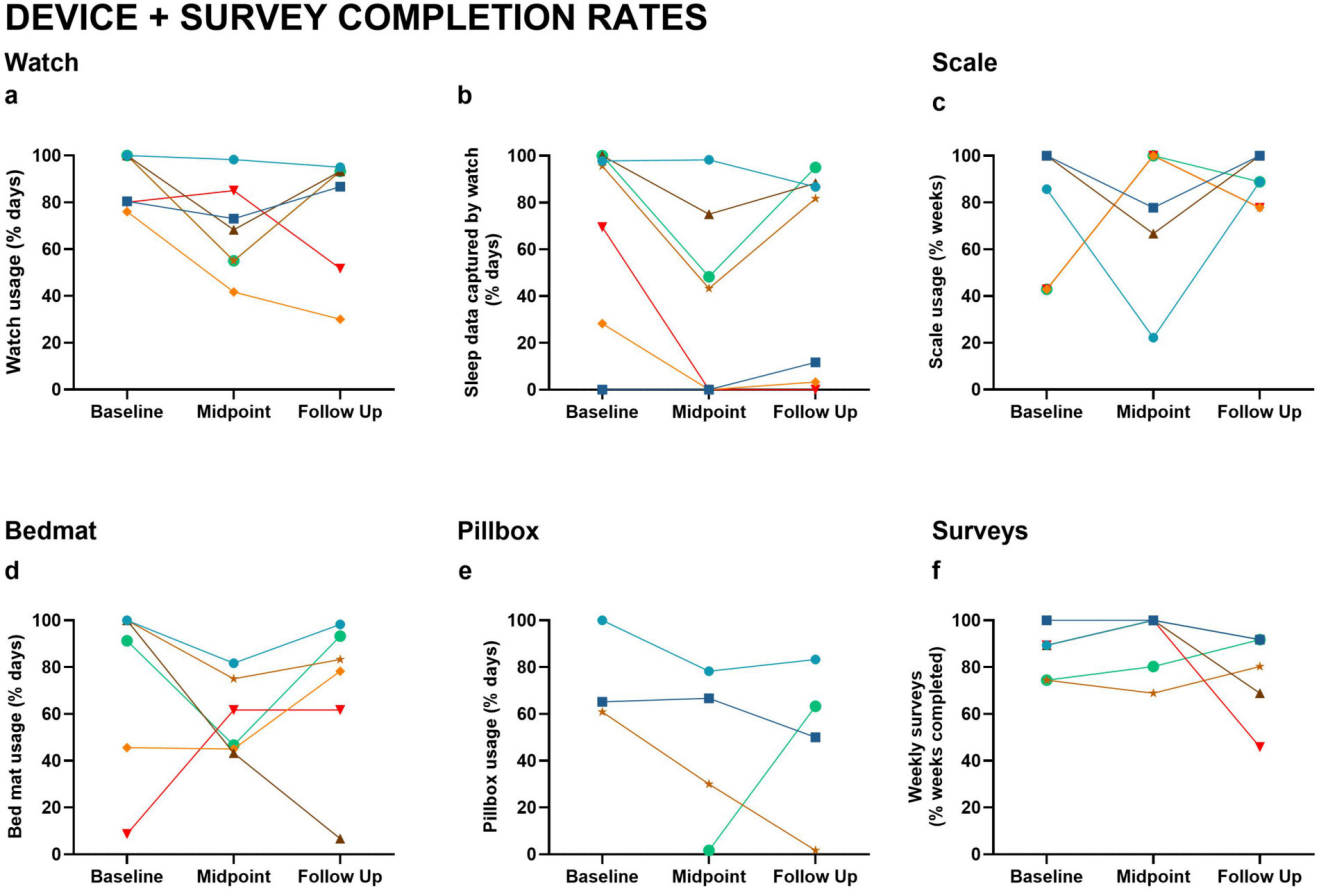
Device and survey completion rates. Usage and data capture rates across Baseline, Midpoint, and Follow-Up include: **(a)** Watch usage (% of days worn); **(b)** Sleep data captured by watch (% of days); **(c)** Scale usage (% of weeks); **(d)** Bedmat usage (% of days); **(e)** Pillbox usage (% of days); **(f)** Weekly survey completion (% of weeks). Lines represent individual participants, with consistent color coding across figures.

The percentage of data obtained for the watch, scale, pillbox, and bed mat varied and was inconsistent across participants and timepoints (Figure 3). Missing data were typically due to problems with the devices or user noncompliance. Survey completion rates were the most consistent over the course of the study, ranging from 74%–97%. Watch usage ranged from 49%–98%. Approximately half of the participants did not consistently wear their watch while sleeping (Figure 3), but of those who did wear their watch, compliance dipped during the study midpoint. Bed mat compliance was similar, ranging from 44%–93% with some user error (e.g., unplugging the device). Scale usage was better, ranging from 66%–93%. Two of the four participants who opted to use the pillbox had consistent data 60%–87% of the time. The pillbox frequently malfunctioned (i.e., the plastic lids were prone to breakage), resulting in a large proportion of missing data.

## Discussion

This study aimed to evaluate the feasibility and acceptability of the ORCATECH platform in a small cohort of diverse individuals in effort to gain insight from their study experiences to inform future research. We found high levels of technology acceptability, with 86% rating their experience as highly satisfactory or satisfactory, suggesting an openness to engaging in digital health research. Nonetheless, consistency in digital data collection was variable across devices and individuals, indicating opportunities to better tailor the technology to meet the needs and interests of the community. In our study, the pillbox received the lowest ratings of usability from participants and had the least viable data collection over the study interval. Based on this finding, the pillbox was omitted from a larger, ongoing study (R01AG077472) This demonstrates the benefit of conducting short-term pilot studies to optimize participant experience and data completeness.

As a secondary aim, we evaluated everyday activity levels using the ORCATECH platform. We generally found no significant changes in the values of activity measurements over the 1-year study, demonstrating the reliability of the platform.

The absence of statistically significant changes in cognitive function from baseline to follow-up was expected given the largely cognitively unimpaired community dwelling cohort. However, depression scores (GDS-15) significantly improved at one-year follow-up, with 71% (*n* = 5) of participants scoring 5 or higher at baseline, suggesting mild depression, compared to 14% (*n* = 1) at follow-up (12). One possible explanation is that our study began in May 2021, when the COVID-19 pandemic was ongoing, and depression was significantly higher in older adults (13). As participants returned to their normal routines and engaged in research activities requiring home visits, their depression symptoms may have lessened through increased social connectedness.

These findings, along with our implementation experience, provide valuable preliminary insights for future research in diverse older adult populations. Our study highlights the need for dedicated, well-trained research staff who conduct weekly data monitoring, provide technology troubleshooting support, and are responsive to participant privacy concerns. For device selection, our preliminary findings suggest digital scales and activity watches have high user acceptance and data completeness, while the challenges with the electronic pillboxes suggested that additional troubleshooting is needed prior to broader implementation.

Our pilot study had several limitations. The cohort was relatively well-educated, which may not reflect broader community attitudes toward technology. Tailored recruitment strategies are necessary to engage participants with varied educational backgrounds in order to improve generalizability. Study design considerations should also include formal assessment of within-household agreement when enrolling couples, comprehensive device orientation sessions, and realistic expectations and statistical powering for compliance variability (20%–30% range observed across devices). Implementation protocols should emphasize privacy protections and installation support, areas where we achieved 100% satisfaction, while building contingency plans for device malfunctions. Finally, outcome measurement should balance comprehensive clinical assessments with participant burden. Our 5-minute weekly surveys showed strong completion rates (74%–97%), suggesting that brief, high frequency data collection may be capable of providing important insights on the course and frequency of fluctuating symptoms (i.e., mood, pain). These evidence-based recommendations can help optimize both participant experience and data quality in future digital health research with underrepresented populations.

In conclusion, the ORCATECH platform for home-based assessment was widely accepted amongst our diverse participants, suggesting its utility for future studies in our South Texas population. Important limitations include the small sample size (*n* = 7) highly educated cohort, and the restriction of single or dual household residence sizes, which limit statistical power and generalizability to the broader South Texas population. Additionally, acceptability was evaluated among participants who elected to enroll in the research study, which may not generalize to the broader community. Despite these limitations, our findings provide important preliminary data on technology acceptability and device performance in this South Texas community, establishing a foundation for larger studies adapted to meet specific population needs. Future directions include evaluating interests and barriers to digital technology research in larger studies, as well as collecting data to optimize the technology to the needs of individuals and the community. More broadly, these findings support the development of home-based digital biomarker platforms that could enable continuous monitoring of cognitive health in diverse older adults, facilitating earlier intervention when disease-modifying treatments are most beneficial.

## Data Availability

The original contributions presented in the study are included in the article/Supplementary Material, further inquiries can be directed to the corresponding author.
